# District of Columbia Veterinary Medical Association

**Published:** 1896-02

**Authors:** 


### DISTRICT OF COLUMBIA VETERINARY MEDICAL ASSOCIATION.

A SPECIAL and general meeting was called by the members on Friday evening, January 10, 1896, in the banquet room, Williard's Hotel. The object of the meeting was to discuss the bill before Congress for the regulation of veterinary medicine and surgery in the District of Columbia, and also to enroll such graduates of the profession who were entitled and desirous of becoming members of the Association. Many veterinarians responded to the call. Dr. C. B. Robinson presided; Dr. Ford, Vice-President; Dr. Acheson, Secretary.

Dr. C. B. Robinson made very clear the advantages which could be gained by a thoroughly harmonious spirit among the members, and pointed out the great need of some form of legislative protection for the public against incompetency in veterinary practice. Dr. J. H. Adamson read a paper on "Organization," and received a vote of thanks for its efficiency. Dr. D. Buckingham also read an able paper on "Reports of Cases," which created a pleasant discussion. Among those present were Drs. C. B. Robinson, Ford, Acheson, Buckingham, Adamson, J. D. Robinson, Pointon, Bushman, Miller, Showalter, Grenfeld, Turner, Barton, Diedrich, and Rome.

